# Seeder Model Challenge of Emerging *Salmonella* Infantis in Broilers: Potential of Organic Acid-Based Feed Additive in Performance and Gut Health

**DOI:** 10.3390/pathogens15020204

**Published:** 2026-02-11

**Authors:** Muhammad Zeshan Aslam, Muhammad Yasin Tipu, Sandra van Kuijk, Asim Aslam, Muhammad Afzal Rashid

**Affiliations:** 1 Department of Pathology, University of Veterinary & Animal Sciences, Outfall Road Lahore, Lahore 54000, Pakistan; zeshanzeshan@hotmail.com (M.Z.A.);; 2 Department of Animal Nutrition, University of Veterinary & Animal Sciences, Outfall Road Lahore, Lahore 54600, Pakistan; drafzal@uvas.edu.pk; 3 Trouw Nutrition R&D, 3800 AG Amersfoort, The Netherlands; sandra.van.kuijk@selko.com; 4 Poultry Research Centre, Casarubious del Monte, 45215 Toledo, Spain

**Keywords:** *Salmonella* Infantis, seeder model, short-chain fatty acids, medium-chain fatty acids, gut health, lesion scoring, antimicrobial resistance

## Abstract

*Salmonella* Infantis is a recognized antimicrobial resistance threat, and in the compromised chicken gut, this pathogen penetrates weakened tight junctions, disrupts the microbiota balance, and triggers inflammation. This study evaluated the potential effects of a feed additive blend comprising short chain fatty acids including coated sodium and calcium butyrate, medium chain fatty acids, and phenolic compounds on broiler gut health and performance under a *Salmonella* challenge using a proprietary seeder model methodology. The foundation, led by a preliminary study as Part A, comprises a negative control and two treatment groups, ran to develop a seeder model, and 1056 day-old Ross 308 broiler chicks were allocated into three groups. On days 5–6, *Salmonella* Infantis 7570 was inducted via seeders in T2 (frozen strain) and T3 (live strain), while T1 remained unchallenged. No significant difference in infection development was observed between T2 and T3 (*p* > 0.05). In T1, *Salmonella* positivity checked via real time polymerase chain reaction (iQ-check II Biorad) remained below 50% at respective time points, indicating that complete isolation in the same room is not feasible. Henceforth, in the main experiment, Part B, 396 male day-old Ross 308 birds were randomly assigned to two further treatments: (1) a positive control group fed a commercial diet, and (2) a treatment group fed the same diet supplemented with 2.5 kg/t of an organic acid-based additive blend (Presan FY, Tilburg, The Netherlands) until day 28. On days 5 and 6, five birds per pen were orally inoculated with 10^9^ CFU/mL *Salmonella* Infantis 7570 obtained via Part A and placed as seeders in both treatments. A borderline tendency for lower *Salmonella* counts was observed in the treatment group on day 19 (*p* = 0.062). The control group had significantly higher lesion scores on day 13 (*p* = 0.0068), with no significant difference on day 19. Body weight was significantly higher in the treatment group on days 11 and 28 (*p* = 0.0157), with no difference on day 39 (*p* = 0.1857). Average daily gain improved significantly between days 11 and 28 (*p* = 0.0234), and feed intake was also significantly higher during this period (*p* = 0.0007). Feed conversion ratio showed a tendency to improve between days 5 and 11 (*p* = 0.0638). Overall, this study reveals that the seeder model can be adopted in *Salmonella* research, and the application of a blend showed a borderline tendency to reduce *Salmonella* counts, however significantly lower the lesion scores, thereby improving gut health in broilers under a *Salmonella* challenge.

## 1. Introduction

Poultry meat has shown high consumption patterns, and in the EU, poultry meat accounts of 40% of the total meat consumption [1]. With increasing demand for poultry and poultry products, the risk of zoonosis, food safety concerns and, most importantly, antimicrobial resistance patterns would have increased parallelly. The urgency to find alternatives to antibiotic growth promoters in poultry production is underscored by AMR’s reported toll of 5 million deaths in 2019, with Asia already facing 1.3 million annual fatalities. Nonetheless, the impact on financial crises and economic losses is of sheer importance related to the culling of the flocks because of increased resistant bacteria incidence [2,3,4]. *Salmonella*, as an enteric pathogen, belongs to Enterobacteriaceae family and Gram-negative genus, with 2600 known serovars, two species (*Salmonella* enterica and *Salmonella* bongori) and clear history of zoonotic losses between humans and animals [5]. *Salmonella* infection is based on preliminary factors like host age, immunosuppression, poor management of flock, stress caused by fluctuation in ambient temperature, infection dose and mixed infection condition [6]. Chicken can be infected with *Salmonella* through (horizontal route) contaminated feed, water and litter and contact with the *Salmonella* reservoir animals like insects and rodents [7]. The vertical or transovarian route is based on *Salmonella* entrance into the egg compartments like the yolk, albumin and vitelline envelope though infection originated in reproductive organs either via the ovary or during the egg’s passage from ovary to the gastrointestinal tract [8]. The *Salmonella* infection dose is directly proportional to the progression of clinical signs in birds, with clinical salmonellosis being more likely to develop in young birds infected with high doses of *Salmonella* Enteritidis [9]. Pathogenicity factors of *Salmonella* are mainly dependent on the virulence genes and plasmids; both are present on the *Salmonella* Pathogenetic Islands, mainly SPI-1 and SPI-2 [10]. salmonellosis, as a zoonotic disease, is well connected to the consumption of chicken’s processed products [11]. In human salmonellosis cases five serovars have gained a target status through laying hens and broilers consumption, namely *Salmonella* Entritidis, *Salmonella* Typhimurium, *Salmonella* Infantis, *Salmonella* Kentucky and *Salmonella* Heidelberg [12]. *Salmonella*-infested food sources can cause non-typhoidal salmonellosis (NTS). Non-typhoidal salmonellosis other than gastrointestinal infection can also be responsible for invasive fetal diseases, osteomyelitis, arthritis and meningitis in humans [13]. The Centre of Disease Control & Prevention and European Food Safety Authority reported around 420 deaths per annum and more than 65, 000 human cases of *Salmonella* in US and EU, respectively, out of which the number of food-borne cases are 6632 (accounts for 21% of total human cases due to zoonosis) [14,15]. *Salmonella* detection in human samples has been observed at different stations in Pakistan, with 50% positive cases in Punjab province, mainly due to an outbreak of *Salmonella typhi* [16]. Independent researchers have also shown the presence of multi-drug-resistant *Salmonella* Infantis in poultry flocks in Pakistan. A total of 54 out of 149 (36%) *Salmonella* isolates of chicken necropsy have been confirmed by targeting serovar-specific gene fragmentation and polymerase chain reaction [17]. Similar types of results, with 33% *Salmonella* Infantis positive isolates, were detected in chicken meat samples through genome sequencing in the Kingdom of Saudi Arabia [18]. The irregular use of antimicrobials to treat salmonellosis has been observed to create antimicrobial resistant strains of *Salmonella*. Thus, the resistance pattern has been converted from single to multiple drug resistance. Recently, extensive drug resistance (XDR-*Salmonella*) and multiple drug resistance (MDR-*Salmonella*) have been seen in some strains [19]. *Salmonella* Infantis has an elevated threat of antimicrobial resistance (AMR) because of its resistance to certain antimicrobials as well as disinfectants and ability to make biofilm and attach to host cells [20]. The CDC [14] reported an outbreak of the *Salmonella* Infantis strain associated with chicken. This strain contained pESI plasmids (plasmids of emerging *Salmonella* Infantis); the importance of these plasmids is containing antimicrobial and multi-drug-resistant virulence genes that help their persistence in poultry and survival in human. Reported first in Israel in 2014, pESI has since been detected in Europe, Asia, Africa, and the American continent [21]. *Salmonella* Infantis prevalence and detection studies around the globe shows the level of zoonotic threat to humans, and therefore strategies and studies are needed to calculate the risk and control the spread. In general, Salmonellosis in chicken showed self-limiting mild gastrointestinal symptoms that lasted between 2 and 7 days. Salmonellosis control strategies are based on two fundamental principles: (a) the reduction in prevalence levels in poultry by means of health, biosecurity, or food strategies and (b) protection against infection in humans. Efforts to reduce transmission of *Salmonella* by food and other routes must be implemented using a One Health approach that gives us a pathway to reduction strategies of *Salmonella* in food and other horizontal routes [22]. In intensive poultry farming, salmonellosis can be fatal to farm economics. Therapeutic antibiotics can be used as curing agents to prevent prevalence in the gut, as well as for growth promotion [23]. NTS infections in chicken continue to remain a significant risk to public health. On-site use of nutritional and non-nutritional strategies and biosecurity measures could help reduce the burden of salmonellosis and antibiotic resistance associated with poultry products [24]. Organic acids from C1-C7 are considered short-chain fatty acids. Inside bacteria, acid works in two ways: reducing the cytoplasmic pH balance by releasing hydrogen ions (in response bacteria depletes energy to balance) and cytoplasmic accumulation of dissociated anions. OA further helps in reducing pH at the proventricular level, which can help in more proteolytic enzyme generation (pepsin), thus allowing better protein digestibility and reduced amino acid availability for pathogens [25]. Medium-chain fatty acids are the group of OA from C6-C12, usually obtained through distillation process of fats. The group consists of caproic (06), caprylic (08), capric (C10) and lauric acids (C12), and it also includes coconut and palm oil. MCFAs, with their affinity to cross the cell membrane of bacteria, are considered antibacterial [26]. It is also stated that the effect of adding MCFAs with SCFAs will give a synergistic effect to control enteric pathogens and enhance gut health [27]. Butyric acid, (C4) produced by fermentation of starch left in cecum and consumed by enterocytes in cranial gastrointestinal tract section [28], plays a part in differentiation and maturation of intestinal epithelial cells and promotes beneficial gut microbes efficacious against pathogenic bacteria such as *Salmonella* spp. and *E.coli.* Phytogenic compounds are the natural plant derivatives known to have efficacy in controlling inflammation, improving animal gut health and halting the enteric pathogen’s growth [29]. Plant extracts can affect the biological functioning of pathogens by disturbing ion exchange process and bacterial biofilms [30] and are the potential priority alternative to replace antibiotics for growth promotion in chicken animal nutrition [31]. Therefore, the current study hypothesizes that the implementation of a practical seeder model and a multi-compound additive treatment based on SCFA, MCFA, phenolic compounds and butyrate could reduce the chicken-borne salmonellosis associated with poultry end-products. Supported by growing demand for non-antibiotic animal gut health products, coupled with concerns over public health, antimicrobial resistance (AMR), and global bans on antibiotic growth promoters, the poultry industry is seeking effective alternatives. The originality of this research depends on evaluating a rightly integrated *S.*Infantis strain for developing the seeder model, macroscopic intestinal lesions, and correct production parameters to offer benefits for broiler gut health and performance. Furthermore, it dispenses the latest insights on *S.*Infantis propagation in different phases. Thus, this article discusses strategic approaches and modeling that demonstrate how a synergistic blend of SCFA, MCFA, butyric acid, and plant extract can enhance gut health and production performance under *Salmonella* challenge conditions.

## 2. Materials and Methods

### 2.1. Part (A)

#### 2.1.1. Housing Protocol

All the parts of this study were conducted at poultry research center Nutreco, Casarrubios del Monte, Toledo Spain. The current study was approved by the Poultry Research Centre Ethical committee as per animal laws and regulations of the European Directive 2010/63/EU. All the birds were randomly allocated to all the pens and treatments, and a pen can be considered as an experimental unit. A total of 1056 day-old broiler chickens, ROSS 308 males, were enrolled in Part A of the study. Birds were housed in groups of 22 birds per pen, with 15/18 pens per treatment in two rooms (Table 1). The freeze strain collected from the source (Masterlab Tres Cantos, Madrid, Spain) is named as 7570 (a), and the live strain collected from the cecal sample of *Salmonella* Infantis birds is named as 7570 (b). Trial is divided into three phases, with phase 1 between 0 and 11, phase 2 between 11 and 28 days and phase 3 between 28 and 35 days. All the birds had *ad libitum* access to feed and water, and the housing management factors like temperature, ventilation, air quality, cleaning protocols and the lighting schedule were set as per the guidelines of Ross 308 broiler management guide [32]. 

#### 2.1.2. Salmonella Inocula Prepation

The *Salmonella* inoculum preparation in this experiment of both previously frozen and live strain (obtained from pre-experiment) types are the same as described. One week prior to inoculation, a cryovial containing the stock culture of *Salmonella* Infantis was thawed and streaked onto a Plate Count Agar (PCA) plate. The plate was incubated at 37 °C for 24 h. A single colony from the PCA plate was subsequently transferred to a Xylose Lysine Deoxycholate (XLD) agar plate and incubated under the same conditions. This process of daily sub-culturing on fresh PCA and XLD plates was continued until the day preceding inoculation to ensure the availability of active and pure colonies. On the day before inoculation, fresh colonies were collected using a sterile loop and suspended in Brain Heart Infusion (BHI) broth. The inoculated BHI was incubated at 37 °C for 20–24 h to obtain an overnight culture with an approximate concentration of 10^9^ CFU/mL, which served as the inoculum for the seeder model.

To verify the concentration of the inoculum, decimal serial dilutions were performed. Aliquots from the dilutions were plated on Petrifilm™ Aerobic Count (AC) plates (3M Company-Neogen, Bridgend, UK (Scotland)), incubated at 37 °C for 48 h, and colony-forming units (CFU) were enumerated at the appropriate dilution level. The *Salmonella* Infantis strain was adapted to rifampicin using the gradient agar technique by selecting colonies able to grow at progressively higher concentrations of the antibiotic, and growth has been tested in the pre-experiment. This extra selective layer opted to purify results and enhance the counting method.

#### 2.1.3. Seeder Challenge Model

Inoculum were developed based on the agar technique to obtain a concentration of 1 × 10^9^ CFU/mL [33]. This infection dose was orally inoculated into seeders and placed on day 5 and 6 into pens of T2 and T3, as in the method explained above. On day 7 (2 days post inoculation (DPI)), cloacal swabs were taken from all seeder birds to test for the presence of *Salmonella* via PCR. On day 13, 19 and 33, all non-seeder birds of randomly selected 5 pens of T2 and T3 were used for *Salmonella* counting by ceca sampling. In the negative control, all pens were swabbed to test for presence of *Salmonella* Infantis.

*Salmonella* counting was performed on the internal standard operating protocol of PRC Nutreco based on preparation of BGA in plates with 1 mL of prepared antibiotic solution (rifampcin 100 μg/mL, MIC = 50) and later extended on different dilutions, i.e., −1, −2, −3, −4, to select the most suitable dilution for counting, which was ideally 10–300 FCUs. Ceca samples were collected in a stomacher bag, transported immediately to the lab and kept in the refrigerator. Extracting cecal content, weighing it and adding buffered peptone water with automatic dilutor (1:10 dilution) enriched with *Salmonella* selective supplement (Bio`-Rad *Salmonella* capsule) were later steps. Once added into the stomacher bag, it was smashed for 30 s and kept at room temperature for 30–60 min before platting. If the *Salmonella* CFUs were less than a bare minimum level on all dilutions, we performed Real time-PCR based on ISO 7218:2024 [34] (IQ-Check *Salmonella* II kit, BioRad, Hercules, CA, USA) to check for positive or negative samples and explained later in PCR section [35].

#### 2.1.4. Dietary Treatment

Birds were fed a commercial, pelleted diet (Table 2), with phase 1 being between days 0–11, phase 2 between days 11–28 and phase 3 between days 28–35. No dietary treatment was applied; all birds received the same diets. 

#### 2.1.5. Growth Performance

For the production parameters on growth performance, the average feed intake and weight gain of all the birds in all the pens were calculated daily and later segregated phase-wise into day 0, 11, 28 and 35. These parameters (BW and FI) were used to calculate feed conversion ratio and feed efficiency phase wise [36]. A routine check for mortality was implemented and calculated regularly to correct growth parameters. 

#### 2.1.6. Real-Time Polymerase Chain Reaction

The objective of the real-time polymerase chain reaction test was rapid detection as well as confirmation of *Salmonella* presence in cecal samples, and it was performed using the iQ-Check *Salmonella* II kit following the known procedure. The following reagents were part of the test performed on the Bio-Rad real-time PCR instrument:

- Oligonucleotides (primers and probes);
- DNA polymerase;
- Nucleotides.

##### Sampling Procedure

The sampling procedure and quantities included cloacal swabs pooled from 10 birds. Samples were collected under sterile conditions and transported to the laboratory for storage under refrigeration (4 °C) and could be stored for up to 96 h before analysis. The sample was placed in a sterile Stomacher bag with a filter. All Purpose Tween 80 broth (APT broth) was added at a dilution of 1:10 to the sample, previously supplemented with RAPID *Salmonella* capsule (1:225 mL). The sample was incubated at 41.5 °C for 19 h. Then, 1 mL of the incubated sample was transferred to a tube containing 9 mL of supplemented APT in a laminar flow cabinet and incubated at 37 °C for 5 h.

##### DNA Extraction

For DNA extraction, the heating block was turned on and set to 98 °C, and 30 min were allowed for temperature stabilization. All reagents were included in the iQ-Check kit. The lysis buffer was placed on a magnetic stirrer for 5 min. A pair of Eppendorf tubes was labeled with sample numbers, and 100 μL of lysis buffer was dispensed into one tube of each pair. In the laminar flow cabinet, 100 μL of the sample was added to the tube containing lysis buffer. Tubes were heated in a heating block at 95 °C for 15 min and centrifuged for 5 min at 15,000× *g* rpm. An aliquot of 50 μL of the supernatant was transferred to a clean, labeled Eppendorf tube. DNA was considered extracted at this point. Samples could be stored at −20 °C for up to one year.

##### RT-PCR Preparation and Amplification

For preparation of the RT-PCR step, reagents for amplification and fluorescence were mixed (MasterMix) in an Eppendorf tube in the laminar flow cabinet. The proportion of mixing was set according to the number of samples, and both positive and negative controls were added with the samples. The MasterMix was used within 60 min of preparation. Forty-five microliters of MasterMix were dispensed into selected wells for controls and samples, and 5 μL of the respective sample was added. Care was taken to ensure that no bubbles formed during plate sealing. The sealed plate was placed in the thermocycler, and the conventional PCR process was executed using the software (CFX Manager Software, Industrial Diagnostic Edition 3593893) provided with kit.

### 2.2. Part (B)

#### 2.2.1. Housing Protocol

An extensive power analysis (Figure S1) was done based on Part A in which it was predicted that with this number of animals a minimal difference of 0.6–0.7 log CFU cecal *Salmonella* could be shown as significantly different (power = 0.80). Henceforward, Part B of experiment was performed similarly to Part A, with the following adjustments. In total, 396 broiler chickens (Ross 308 Males) were included, housed in 9 pens with 22 birds each per treatment. Similarly to Experiment A, three feeding phases were included; however, the total length of the study was extended to 42 days. Additionally, the seeder birds were housed separately and fed the control diet until inoculation, after which they were placed in their respective treatment pens.

#### 2.2.2. Seeder Challenge Model and Salmonella Challenge Design

*Salmonella* Infantis 7570 has been prepared in the same way as in Experiment A treatment 2 and was used for oral inoculation according to the methods described in Experiment A, after which the seeders were placed into the respective pens. On days 13, 19 and 40, four non-seeder birds per pen were selected for cecal *Salmonella* counting via plating as in the method explained above.

#### 2.2.3. Dietary Treatment

Birds were fed a pelleted, commercial diet (Table 2) and divided into three phases: (1) Phase 1, day 0–11; (2) Phase 2, day 11–28; (3) Phase 3, day 28–42. On day 39, a feed withdrawal process was applied before the final sampling on day 40.Two treatments were applied: positive control diet without feed additive; treatment with additive diet, which was the positive control diet with Presan FY named as PST (2.5 kg/t, Selko, Tilburg, The Netherlands) added in feeding phases 1 and 2; and no additive in feeding phase 3. 

#### 2.2.4. Measurements

Growth performance was measured according to the same methodology as described for experiment A [37]. Measurements in Experiment B took place on day 0, 5, 11, 28 and 42. Macroscopic gut health scoring points are mainly divided into the proximal and distal gastrointestinal tract named as (i) Ballooning, (ii) Inflammation, (iii) Flaccid, (iv) Translucent, (v) Content, (vi), Undigested Particles, and the proximal part includes the duodenal loop, jejunum and Meckel’s diverticulum marked as distinction between distal part. [38] Distal parts include remaining ileum, cecum, and cecal tonsils. Mean Lesion Score was calculated for both control and treatment on day 13 and day 19 on 5 non-seeder birds per treatment and compared for results. The same animals’ cecal tonsils were used for *Salmonella* counting in Part B of the experiment to keep corelate the results b/w gut health scoring and *Salmonella* counting. 

#### 2.2.5. Statistical Analysis

All the data of Part A and B has been statistically analyzed on randomized complete block design by SAS Studio version 9.0.The quantitative *Salmonella* counts and growth performance were analyzed By PROC MIXED, keeping treatment as a fixed factor while mortality, qualitative *Salmonella* counts, and lesion scoring were analyzed through PROC GLIMMIX, as these factors are based on non-normal distribution. One-way ANOVA was applied in all procedures to analyze differences in *Salmonella* counts, growth performance and gut health scoring. Later, Tukey’s Post Hoc test (HSD) was used to distinguish the difference in *p* value (<0.05) as significant and *p* value (<0.1) as tendency. 

## 3. Results and Discussion

### 3.1. Part (A) 

#### 3.1.1. Salmonella Infantis Strain Selection

##### Unchallenged Negative Control

A total of five birds were sampled by cloacal swabs from each pen of the unchallenged negative control on day 13, 19 and 33. A reverse transcriptase polymerase chain reaction based on cloacal swab samples on day 13, 19 and 33 had shown 7/18, 7/18 and 3/18 positive birds, respectively, with same serovar of *S.*Infantis, explaining no inclusion of the negative control in the main experiment referred to as Part B (Table 3), which was later confirmed through the *Salmonella* culturing technique using the standard operating procedure of Trouw Nutrition Research and development facilities based on peptone water and extension by BGA method. In contrast, Peter et al. [39] showed that an untreated and unchallenged control group remained negative starting from day 0 until 16 weeks in hens for *S. typhimurium* in experimental studies. In the current set up of this study, a negative control is not possible as it spreads horizontally through the farm, and a negative control should be run in a separate room to avoid *Salmonella* transmission. This preposition is in line with *Salmonella* colonization studies of L. Revolledo and I. Gantois in which both broilers and layers were used to run a negative control in separate rooms [40,41].

##### Challenged *Salmonella* Infantis 7570a vs Challenged *Salmonella* Infantis 7570b Strain

As discussed, SI 7570a and SI 7570b are obtained by frozen sample and live sample, respectively, which were compared to identify the best way to obtain *Salmonella* Infantis for a successful model. It is important to select the right strain to obtain 10^9 colony-forming units/milliliter concentration with a propagation pattern mimicking the exaggerated practical conditions to show differences. Seeders placed on day 5 and 6 with infection dose in T2 and T3, upon testing for *Salmonella* presence through qualitative cecal swab test on day 7 (2 Dpi), have shown >75% positivity on all time points. 

The qualitative analysis of *Salmonella* presence on day 13, 19 and 33 of T2 (7570a) had shown a 100%, 100% and 88% positive ratio, respectively; similar results were been seen in T2 (7570b) with 100%, 100% and 92% positive results on respective days (Table 4).

These type of results are expected because *Salmonella* Infantis has more intensive intestinal colonization than other serovars because the predominant route of transmission is horizontal [42]. *Salmonella*-positive bird reduction had been observed after 28 days in both treatments, showing the endorsement of the self-limitation pathogen behavior and self-body recovery at 14 days post infection, since the highest production of antibodies have been observed in same time frame in other *Salmonella* studies in chicken [42,43]. RT-PCR detection has shown no significant difference between the two treatments, concluding there is no difference in infection propagation of the two strain types in in vivo studies. Statistical analysis of data obtained through cecal *Salmonella* counts shows non-significance between T2 and T3 (*p* > 0.05) on all time frame points (Table 4). Earlier studies of stored *Salmonella* Infantis serovar 1326 at −20 °C have also shown successful infection propagation and elevated immune response by avian macrophages [44]. Based on the results of qualitative studies through RT-PCR Detection, the seeder model was considered successful for both strain types, 7570a and 7570b, regardless of storage, as results had shown no difference in *Salmonella* propagation on day 13 (*p* = 0.3227), day 19 (*p* = 0.9723) and day 33 (*p* = 0.1514) in non-seeder birds. There is also no significant difference (*p* > 0.05) between overall mortality and morbidity noted from day 0–35 in T2 and T3 (Table 4). Due to the labor intensity issue, *Salmonella* Infantis strain 7570a (SI 7570a T2) has been selected for further investigation in the main experiment. 

### 3.2. Part (B)

#### 3.2.1. *Salmonella* Infantis Colonization in Caeca: A Qualitative and PCR Detection of *Salmonella*

**Figure 1:**
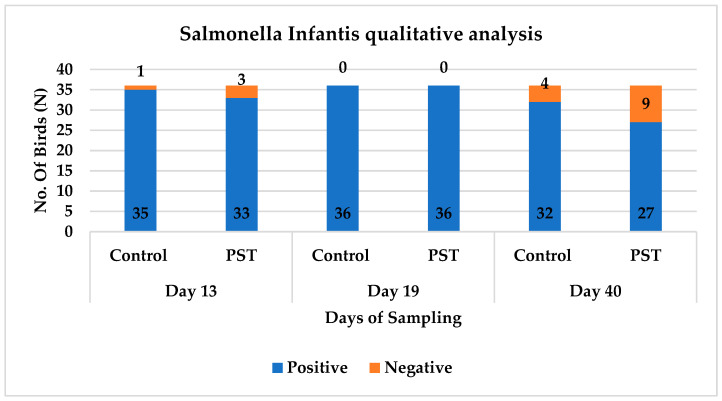
Qualitative analysis of *Salmonella* Infantis colonization in broiler chickens across different sampling days (day 13, 19, and 40). Bars represent the number of birds testing positive (blue) and negative (orange) for *Salmonella* Infantis in both control and feed additive treatment PST groups.

The *Salmonella* Infantis pathogenic dose in seeders placed in all groups was log 9 CFU/mL. The results have shown no significance, as the number of *Salmonella* Infantis positive birds in the challenge control group were 35, 36 and 32 out of 36 birds, while in PST group the number of positive birds is 33, 36 and 27 (*p* > 0.1) on day 13, 19 and 40, respectively (Figure 1). Victoria Drauch, in her study, stated better susceptibility of *S.*Infantis shedding after induced infection in Ross 308 Vs Hubbard 757 broilers (only 1/25 birds are positive in Hubbard JA 757) and discussed the breed dependency against *Salmonella* infection, but breed-related host immune response and association factors have to be further investigated because the same study revealed no significant differences in *Salmonella* colony counts in cecum between different breeds [45]. Relative in vitro studies of pESI-like plasmid containing *Salmonella* Infantis strains such as the one used in this experiment comprise enhanced virulence factors and better persistence in poultry host tissues and might explain the answer to the minor response against PST treatment [46]. Maria Braukmann stated the invasion and uptake of *S.*Infantis in primary macrophages as weak survival serovar; despite this, the findings of this experiment may explain that the PST type of organic acid-based combination mainly affects endogenous avian epithelial cells as an energy source that works as an agonist of free fatty acids and inhibits the pro-inflammatory response, which only leads to less inflammation in the gut [44]. Cecal colonization of *S.*Infantis in the positive control group compared to PST is 4.71 vs 4.43, 3.58 vs 2.86 and 1.74 vs 1.46 log CFU/mL on day 08 dpi (day 13), 14 dpi (day 19) and 35 dpi (day 40), respectively (Table 5). *Salmonella* Infantis concentration tended to be lower in the PST group on day 19 (*p* < 0.1), although no significant differences were found on day 13 or day 40 (Table 5). The above-mentioned scenario of marginal counts in the grower phase due to SCFAs, specially butyrate, MCFAs and essential oil combination, has been endorsed by Makled MN [47] in his comparative studies; using an organic butyrate combination displayed beneficial impacts on gut microbiota of broiler chickens at day 21 through the reduction in cecal aerobic bacteria, ileal Enterobacteriaceae count and an increase in ileal lactobacilli count compared to the control group. Furthermore, Shaaban S. Elnesr [48] and Van Immerseel [49] also separately briefed an indirect reduction in acid-intolerant *Salmonella* spp. counts in carcass and eggs by use of organic acid combinations with and without butyrate. 

#### 3.2.2. Gut Health Scoring 

Gut health scoring, also called Gut lesion scoring and dysbacteriosis scoring, is a macroscopic scoring system developed by MD. De Gusseum [50] that is used widely on poultry farms in live and uniform animals to analyze the visual lesions/dysbacteriosis pattern based on ten binary factors. The gut health scoring method has been performed blindly in a separate room by trained staff (Credential ID EBP/N/2020/0097) on five randomly selected birds per treatment in the positive challenge and PST group on day 13 and day 19 to visualize macroscopic changes in the gut. The mean lesion scores on day 13 in the PST group, with mean value 2.4 (±0.55), have shown significant reduction relative to the challenge control mean lesion score of 5.2 (±0.56) (*p* = 0.0068) (Table 6)). Wongkuna S [51] studied the improvement in overall gut health due to less tissue damage and inflammation associated with reduction in *Salmonella* species in the GIT of chicken. Another study by Lonneke Onrust [52] explained *Salmonella* as SRB (sulphate reducing bacteria), which compete for lactate in the gut with butyrate-producing bacteria. Thus, an increase in *Salmonella* spp. In the GIT can imbalance microbiota and affect gut health by inducing a more pro-inflammatory response. There was no difference observed on day 19 (*p* > 0.05). Aljumaah MR [53] explained the same results of ileal histomorphometry on day 40 in a study of *Salmonella* Typhimurium challenged broilers supplemented with organic acids; the difference between unchallenged treatment and challenged treatment of *Salmonella* Typhimurium was non-significant in terms of villi length, width and surface area. 

#### 3.2.3. Growth Performance in Challenged Seeder Model 

**Figure 2:**
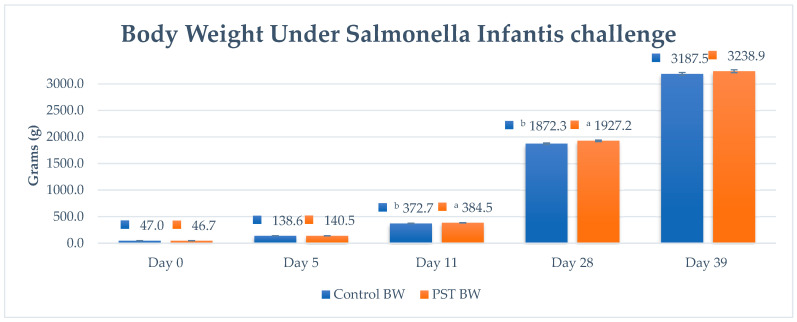
Body weight (grams) (± standard error) on different time points. Bars represent the body weight (blue) positive challenge control and (orange) PST treatment. Different superscripts per column indicate significant differences.

The growth performance was analyzed based on average values phase-wise for body weight (BW), daily gain (ADG), feed intake (FI) and feed conversion ratio (FCR). The mean body weight (g) in the treatment group was 46.7 g, 140.5 g, 384.5 g, 1927.2 g and 3238.9 g on day 0, 5, 11, 28 and 39 respectively, showing a significant difference at day 11 and 28, mainly in grower phase (*p* < 0.05), versus the lower BW in control group, i.e. 372.7 g and 1872.3 g (Figure 2). No significant differences have been seen at other sampling points. Similar non-significant results in the weight gain (WG) and average daily gain (ADG) have been explained in a similar type of study done by Pratima Adhikari [54] which compared *Salmonella* colonization in chicken between a positive control and a treatment group fed short-chain fatty acids. Hu Z. in his studies reported that no significant differences were observed in *Salmonella* Enteritidis challenge in broilers, specifically on average body weight from day 0–39, between challenged control vs three linear-incremented treatments of Essential oils and OA combination (300 g/Mt, 600 g/Mt and 800 g/Mt) (*p* = 0.471, *p* > 0.05), while the current study shows a significant difference in BW only in the grower phase [30].

**Figure 3:**
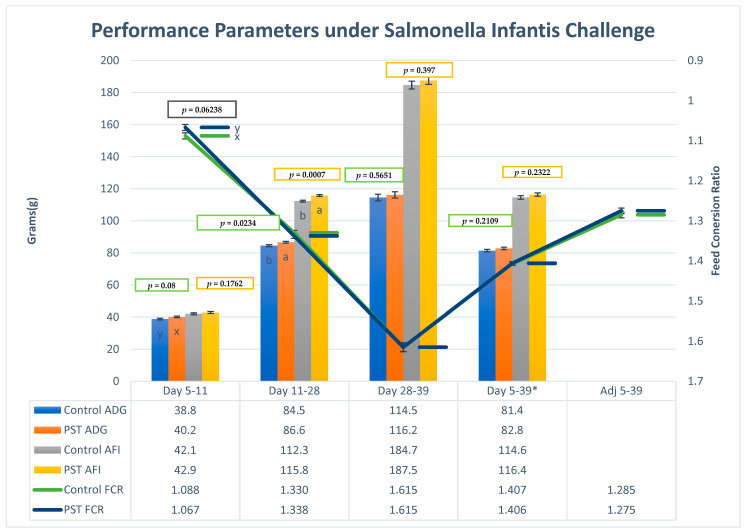
Average daily gain per bird on different days and total flock length, expressed in gram per bird per day (± standard error). * Day 5 = 0 Post infection day, x,y tendency to be different (*p* < 0.1), ^a,b^ indicate significant differences (*p* < 0.05). Average feed intake of all phases and total cycle length, expressed in gram per bird per day (± standard error), * Day 5 = 0 Post infection day, ^a,b^ indicate significant differences (*p* < 0.05). Feed conversion ratio of all phases, total cycle length and adjusted feed conversion ratio. Day 5 = 0 days post infection, x,y tendency to be different (*p* < 0.1).

The average daily gain (ADG) per bird has shown a tendency and significant difference in same pattern from day 5–11 in starter (*p* < 0.1) and 11–28 in grower, resulting in 86.6 g per day (*p* = 0.0234), respectively, with no significant differences observed in ADG at other sampling points in the broiler cycle (Figure 3). The mean of daily feed intake observed relatively the same scope of significance (*p* < 0.05, *p* = 0.0007) in the grower phase (day 11–28), and no significant differences were observed (*p* > 0.05) in any group in all phases between treatment and the challenge control group (Table 3) on sampling days, as well as in total flock length between day 5–39, respectively. The feed conversion ratio is starter (day 5–11) borderline tendency to be lower (*p* < 0.1) in phases and total flock length (*p* > 0.1) between both groups on all sampling days (Figure 3). The adjusted FCR has also shown no significant difference between 0 and 39 days; adjusted FCR is mitigating the effects of abnormal mortality and animals used for gut health scoring. Although adjusted feed conversion ratio (FCR) calculations were applied to mitigate the impact on production metrics, the reliability of performance outcomes could be further strengthened in future experiments by eliminating this factor through increasing birds pool per treatment.

- Adjusted FCR

*FCR_adj_* = *FCR_actual_* − 0.1775 × (*final BW_actual_* − 2.5 kg)

## 4. Conclusions

Negative controls in *Salmonella* Infantis colonization experiments should be maintained in separate rooms under strict biosecurity to prevent pathogen transmission. Nonetheless, the maintenance of a true negative control in the present setting was likely influenced by horizontal transmission; this inherent limitation should be considered when interpreting colonization patterns. The propagation and growth of the same *S*.Infantis strain obtained from two inoculation methods (frozen and live) showed no significant difference in birds, indicating that an equivalent infective dose can be achieved at any point in the broiler cycle. The hypothesis that gut health in chickens can be improved by supplementing a combination of short-chain fatty acids (SCFA), medium-chain fatty acids (MCFA), coated butyrate, and phenolic compounds during a *Salmonella* challenge was supported primarily during the grower phase, with minimal differences observed as the production cycle progressed. Although cecal *Salmonella* counts numerically curtailed in the treated group during the grower phase compared to the challenged control, possibly due to the use of a high infective dose of *S*.Infantis, further investigation is warranted regarding both the challenge dose and optimization of additive composition. Performance data indicate that the additive combination improved gut integrity and homeostasis and exhibited a biological tendency to yield better results during the starter and grower phases under *Salmonella* challenge, reflected in higher body weight, feed intake, and average daily gain. The diminished effect observed in the finisher phase may be attributable to additive withdrawal. Collectively, these positive effects on intestinal robustness and performance suggest that this feed additive has potential as an alternative to antibiotic growth promoters (AGPs) in chickens under a *Salmonella* challenge. In conclusion, although pESI plasmid detection in the current *S.*Infantis strain 7570 was not undertaken here, its assessment could be valuable for future research on antimicrobial resistance within the food chain. Both frozen and live forms of the strain can be effectively used to study salmonellosis in chickens. Supplementation with a feed additive comprising a selected blend of organic acids, coated butyrate, MCFAs, and phenolic compounds may modulate cecal *Salmonella* Infantis counts and growth performance under challenge conditions while significantly improving gut health.

## 5. Limitations

The integrity of the negative controls was influenced by horizontal transmission, a typical occurrence in *Salmonella* challenge models.

## Figures and Tables

**Table 1:** Study design and birds’ allocation in Part A and Part B.

Treatment Name	Replicates per Treatment	Animals per Replicate/Pen	Animals per Treatment	Seeders
**Part A**				
**Negative Control-T1**	18	22	396	0
**Challenge control 7570 (a)-T2**	15	22	330	75
**Challenge control 7570 (b)-T3**	15	22	330	75
**Total**	48		1056	150
**Part B**				
**Positive control**	9	22	198	45
**PST group**	9	22	198	45
**Total**	18		396	90

**Table 2:** Composition and calculated nutritional analysis of Part A & Part B.

	Part A					
	Negative Control, T2, T3					
	Part B					
Items	Positive Challenged Control			PST Treatment		
	Starter	Grower	Finisher	Starter	Grower	Finisher
Ingredients						
Wheat	48.95	54.85	58.35	48.95	54.85	58.35
Soyabean Meal 47%	33.39	28.09	24.58	33.39	28.09	24.58
Corn	10.00	10.00	10.00	10.00	10.00	10.00
Soya Oil	3.51	3.67	4.28	3.51	3.67	4.28
Calcium carbonate	1.38	1.15	0.91	1.38	1.15	0.91
Monocalcium phosphate	0.98	0.40	0.10	0.98	0.40	0.10
NaCl Fine	0.17	0.15	0.16	0.17	0.15	0.16
Sodium carbonate	0.25	0.25	0.24	0.25	0.25	0.24
DL Methionine 99%	0.29	0.29	0.26	0.29	0.29	0.26
L Lysine HCL 98%	0.22	0.28	0.26	0.22	0.28	0.26
L Threonine 98%	0.10	0.12	0.11	0.10	0.12	0.11
L Valine 96.5%	0.05	0.06	0.06	0.05	0.06	0.06
Phytase 5000 IU	0.10	0.10	0.10	0.10	0.10	0.10
Xylanase+ Beta-glucanase	0.10	0.10	0.10	0.10	0.10	0.10
Premix 1.0 *	0.50	0.50	0.50	0.50	0.50	0.50
Totals	100	100	100	100	100	100
PST *	-	-	-	0.25	0.25	-
Totals	100	100	100	100.25	100.25	100

* Premix 1.0, Vit A total IU10000.0, Vit D3 total IU 2500.0, Vit E-ac total IU 50.0, Vit B1 total (as thiamine) mg 2.0, Vit B2 total mg 6.0, Vit B6, total (pyridoxine HCl) mg 4.0, Niacin total mg 40.0, Panto acid tot mg 10.0, Biotin total mcg 150.0, Vit B12 total mcg 25.0, Chol-Cl total mg 300.0, Chol-OH total mg 260.4 and macro minerals. * PST PRESAN FY: Na and Ca target release butyrate, sorbic acid, maltodextrin, distilled palm and coconut fatty acids, Silicic acid, vegetable oil of soya, vegetable fat of palm, acacia. Dry matter 919.80 g, ME Broiler 2019 (Kcal) 2925.0, Crude Protein 196.6 g.

**Table 3:** Qualitative detection of *Salmonella* Infantis in unchallenged negative control birds at different sampling days. Birds were sampled by cloacal swabs from two sections (9 pens/section) on day 13 (D13), day 19 (D19), and day 33 (D33).

	Section 1 Positive Birds/9 Pen	Section 2 Positive Birds/9 Pen	Total Positive Birds/18 Pen
D13	3	4	7
D19	1	6	7
D33	0	3	3

**Table 4:** Qualitative *Salmonella* presence, PCR detection of *Salmonella* Infantis colony-forming units, expressed in log cfu/mL (± standard error) and mortality and morbidity results, expressed in percentage (± standard error).

Treatment	Name	Day 13			Day 19			Day 33		
		Birds- Positive	Birds- Negative	Mean (± Standard Error)	Birds- Positive	Birds- Negative	Mean (± Standard Error)	Birds- Positive	Birds- Negative	Mean (± Standard Error)
2	SI 7570a	75	0	4.47 (±0.184)	75	0	3.51 (±0.144)	65	9	1.35 (±0.118)
3	SI 7570b	75	0	4.73 (±0.185)	75	0	3.50 (±0.144)	68	6	1.61 (±0.132)
	*p*-value			0.3227			0.9723			0.1514
		**Mortality**			**Morbidity**			**Total**		
1	Negative control	2.7 (±0.90)			1.0 (±0.40)			3.4 (±1.11)		
2	SI 7570a	4.0 (±1.34)			1.0 (±0.40)			5.4 (±1.69)		
3	SI 7570b	4.0 (±1.31)			0.9 (±0.37)			4.6 (±1.49)		
	*p*-value	0.3872			0.9582			0.3762		

**Table 5:** *Salmonella* Infantis PCR detection expressed in colony forming units log cfu/mL ((± standard error).

Cecal *Salmonella* Infantis Counts on Time Points				
Treatment	Description	Day 13	Day 19	Day 40
Positive Control	Positive Control	4.71 (±0.462)	3.58 (±0.254)	1.74 (±0.228)
PST-Group	Additive treatment	4.43 (±0.462)	2.86 (±0.254)	1.46 (±0.225)
*p*-value		0.6752	0.0624	0.3929

**Table 6:** Mean gut health scores of cranial and caudal GIT parts expressed as average of binary scores (0 no lesion, 1 lesion present) of each bird at sample point time (± standard error).

Tre*	Description	Day	Zone	Bal*	Inf*	Trans*	Flac*	Cont*	Undig Pr*	Sum	Mean
1	Positive Challenge Control	13	Proximal	1	2	1	3	3	-	26	5.2 (±0.55)
		13	Distal	1	5	4	2	2	2		
2	PST	13	Proximal	0	1	0	0	1	-	12	2.4 (±0.55)
		13	Distal	1	1	3	2	1	2		
P											0.0068
1	Positive Challenge Control	19	Proximal	1	3	3	5	2	-	27	5.3 (±0.54)
		19	Distal	1	1	5	4	0	2		
2	PST	19	Proximal	0	3	1	4	2	-	20	4.0 (±0.54)
		19	Distal	0	2	3	5	0	0		
P											0.1290

Tre* = Treatments, Bal* = Ballooning, Inf*= Infllamation, Trans* = Transleucent, Flac* = Flaccid, Cont* = Abnormal content, Undig Pr* = Undigestible particles in distal intestinal tract, P = *p*-value or probability value, underline = significant value.

## Data Availability

The original contributions presented in this study are included in the article/Supplementary Materials. Further inquiries can be directed to the corresponding author.

## Supplementary Materials

The following supporting information can be downloaded at https://www.mdpi.com/article/10.3390/pathogens15020204/s1, Figure S1. Power analysis to determine the number of animals required (Exp. Part A).

## Abbreviations

The following abbreviations are used in this manuscript:

ABW	Average Body Weight
ADG	Average Daily Gain
AGP	Antibiotic growth Promoter
AMR	Antimicrobial Resistance
ANOVA	Analysis of Variance
BGA	Brilliant Green Agar
C4	Butyric Acid/Butyrate
CDC	Centers for Disease Control and Prevention
EFSA	European Food Safety Authority
EPEF	European Poultry Efficiency Factor
EU	European Union
FCR	Feed Conversion Ratio
FI	feed intake
GIT	Gastrointestinal Tract
MCFA	Medium Chain Fatty Acids
NTS	Non-Typhoidal Salmonellosis
OA	Organic Acids
PCR	Polymerase Chain Reaction
PST	(Presan feed additive treatment group)
SCFA	Short Chain Fatty Acids
WG	Weight Gain
XDR	Extensively Drug-Resistant
