# Supplementary figures and images for: Seeder Model Challenge of Emerging Salmonella Infantis in Broilers: Potential of Organic Acid-Based Feed Additive in Performance and Gut Health
Source: Pathogens. 2026 Feb 11;15(2):204. doi: 10.3390/pathogens15020204 (PMC12942953; doi:10.3390/pathogens15020204)

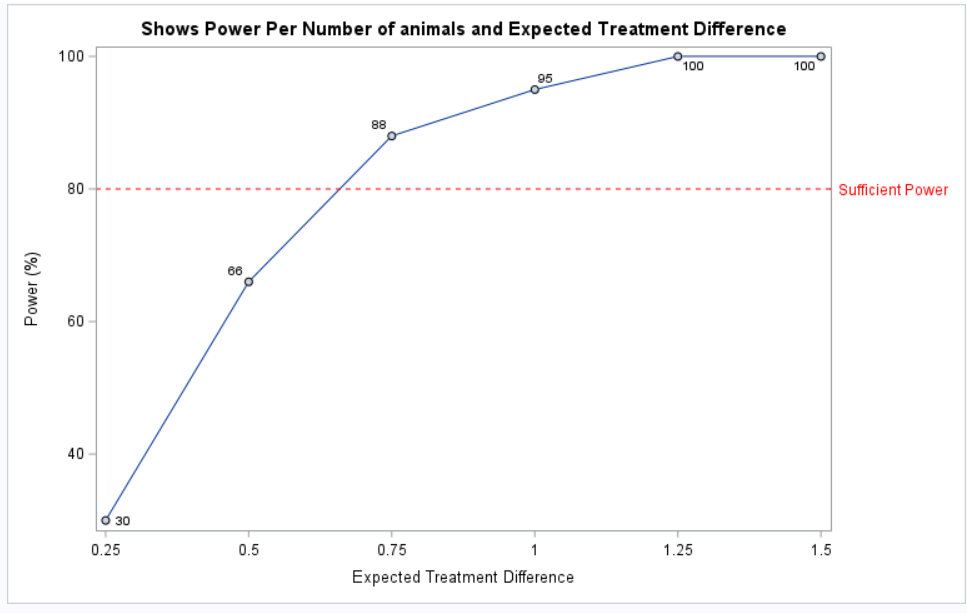

Supplement: Supplementary file 1 [file pathogens-15-00204-s001.zip › Figure S1.png]
